# Postpartum cerebral arterial dissections: Clinical features and treatment

**DOI:** 10.1097/MD.0000000000027798

**Published:** 2021-11-24

**Authors:** Chun-Yun Ruan, Bu-Lang Gao, Hong-Li Pang, Kun Zhang, Yao-Hui Zhang, Li-Ping Wei, Tian-Xiao Li, Zi-Liang Wang

**Affiliations:** a Luoyang Central Hospital, Zhengzhou University, China; b Henan Provincial People's Hospital, Zhengzhou University, China.

**Keywords:** carotid arterial dissections, complications, postpartum, vertebral arterial dissections

## Abstract

Postpartum cerebral arterial dissections are rare, and the clinical features, diagnosis, and treatment approaches are not clear to many physicians. This study was to investigate the clinical features, diagnosis, and treatment of postpartum cerebral arterial dissections.

One patient with postpartum cerebral arterial dissections enrolled in our hospital was analyzed. All patients with postpartum cerebral arterial dissections retrieved from the PubMed were also included in this study and analyzed.

A total of 45 patients with postpartum cerebral arterial dissections were retrieved including our case, with an age range of 24 to 44 years (mean 34). Thirty-six (80%) patients were older than 30 years of age (mean 35). There were 17 cases of cesarean section, 14 cases of natural labor, and 14 cases whose delivery modes were not reported. The clinical symptoms included headache in 35 cases (78%) and neck pain in 14 (31%). The symptoms occurred at a mean time of 11 days (range 0-53 days) following delivery. Among 45 patients, arterial dissections involved unilateral carotid or vertebral artery in 29 cases (64%), bilateral carotid or vertebral arteries in 8 (18%), 3 arteries in 3 (7%), and all bilateral carotid and vertebral arteries in 5 (11%). Fourteen (31%) patients were treated with antiplatelet agents, 27 (60%) with anticoagulation, 7 (16%) with both antiplatelet and anticoagulation medications, and only 2 (4%) with stent angioplasty. The prognosis was complete recovery in 30 (86%) patients and mild focal neurological symptoms in 5 (14%).

Postpartum cerebral arterial dissections are rare, and correct diagnosis relies on imaging examination. Prognosis is usually favorable in patients with early diagnosis and prompt treatment.

## 1 Introduction

Young strokes (18-50 years old) account for about 10% to 15% of all strokes,^[1,2]^ and the risk factors are quite different from those of middle-aged and elderly strokes, including oral contraceptives containing estrogen, migraine aura, pregnancy, patent foramen ovale, genetic correlation, antiphospholipid syndrome, sickle cell disease, carotid or vertebral artery dissection, myofibrous dysplasia, Moyamoya disease, and arteritis.^[3,4]^ Dissections can occur in both intracranial and extracranial arteries, and the incidence of extracranial carotid or vertebral arterial dissections is approximately 2.6/100,000 per year. Postpartum cerebral arterial dissections are much rarer, accounting for only 6% of spontaneous arterial dissection in women under the age 50 years.^[5]^ Headache and neck pain are the most common symptoms in postpartum cervical carotid and vertebral artery dissections, accounting for 60% to 90% of cases.^[6,7]^ However, headache is also commonly presented in puerpera after delivery and may be caused by muscle strain, sleep deprivation, dehydration, or preeclampsia. Some more severe and life-threatening conditions may also cause postpartum headache including subarachnoid hemorrhage, posterior reversible leukoencephalopathy, and cerebral venous sinus thrombosis. Carotid dissection is a much rare cause of pregnancy-associated stroke, which is more common in patients with cardioembolic diseases (valvular heart disease, atrial fibrillation, peripartum cardiomyopathy, and patent foramen oval), intracranial venous (dural sinus thrombosis and cerebral venous thrombosis) or arterial diseases (intracranial aneurysms and arteriovenous malformations).^[8–12]^ Risk factors for maternal stroke include traditional modifiable cardiovascular risk factors (hypertension, obesity, smoking, and hyperlipidemia), hypertensive diseases of pregnancy, migraine, infection, and hypercoagulable states.^[12–15]^ Nonetheless, postpartum cerebral arterial dissections are a common nonnegligible risk factor for ischemic stroke even in healthy postpartum patients.^[6,16,17]^ Thus, it is crucial for the clinicians to identify the presentations, diagnosis, and management of postpartum cerebral arterial dissections because delayed diagnosis may cause severe neurological deficits and even death. In this study, 1 patient with postpartum bilateral cerebral arterial dissections was presented and analyzed with review of the current literature regarding patients with postpartum cerebral arterial dissections.

## 2 Materials and methods

This retrospective 1-center cross-sectional study was approved by the ethics committee of Henan Provincial People's Hospital (20201252). The patient in our hospital had given written informed consent to participate and to publish the relevant data in the journal. A literature search was conducted on the online PubMed for all cases of postpartum cerebral arterial dissections reported between 1966 and 2020 using the following terms for search: “postpartum artery dissection”, “postpartum cervical dissection”, “postpartum dissection”, “postpartum vertebral dissection”, “postpartum carotid dissection”, “pregnancy artery dissection”, and “pregnancy cervical dissection”. Literature search was also performed in the Chinese medical network of VIP, CNKI, and Wanfang databases. In addition, 1 case in our hospital with postpartum cerebral dissections was also recorded and analyzed.

### 2.1 Statistical analysis

Statistical analysis was performed with the SPSS software package (IBM, Chicago, IL), and the mean, median, and standard deviation were computed.

## 3 Results

The literature search in PubMed and in Chinese medical network databases yielded 30 articles including 44 patients with postpartum cerebral arterial dissections^[6,16–43]^ (Table 1). Including the case from our hospital, there were a total of 45 patients, with an age range of 24 to 44 years (mean 34). Thirty-six (80%) patients were older than 30 years of age (range 30-44, mean 35). There were 17 cases of cesarean section, 14 cases of natural labor, and 14 cases whose delivery modes were not reported. The clinical symptoms of postpartum cerebral arterial dissections were similar to those of other cerebral arterial dissections, including headache in 35 cases (78%) and neck pain in 14 (31%). Twenty-six (58%) patients were complicated with focal neurological symptoms. The symptoms occurred within 6 weeks after delivery in all patients except 1 who had the symptoms 53 days later, with the mean time of 11 days (range 0-53 days). Only 6 patients (13%) had prolonged second stage of labor.

**Table 1:** Clinical feature and treatment of patients with postpartum cerebral arterial dissections.

CN	RN	Age (yr)	Time from delivery (d)	Symptoms	Delivery mode	Arteries involved	Treatment	Prognosis
1	^[43]^	44	6	Headache and right mild hemiplegia	Prolonged second stage of labor and cesarean section	LICA	No	Mild right upper limb weakness and aphasia
2	^[26]^	36	14	Headache and right mild hemiplegia	Cesarean section	LICA	Anticoagulation	Not reported
3		34	6	Headache and aphasia	Natural labor	LICA	Anticoagulation	Not reported
4		26	9	Right retroorbital pain and dysarthria	Natural labor and prolonged second stage of labor	RICA	Anticoagulation	Not reported
5		34	14	Headache, neck pain and right facial numbness Left weakness, dizziness, and right Horner syndrome	Natural labor	RVA	Anticoagulation	Complete recovery
6	^[35]^	38	5	Headache	Natural labor	RICA	Anticoagulation	Complete recovery
7	^[34]^	37	9	Headache, Horner syndrome and aphasia	Prolonged second stage of labor and cesarean section	BICAs+BVAs	Anticoagulation	Not reported
8	^[18]^	35	9	Headache and neck pain	Cesarean section	RICA	Anticoagulation	Complete recovery
9	^[31]^	32	7	Headache and Horner syndrome	Prolonged second stage of labor and natural labor	RICA	Anticoagulation	Complete recovery
10	^[30]^	32	12	Headache and right mild hemiplegia	Cesarean section	RICA+BVAs	Antiplatelet	Not reported
11	^[42]^	36	4	Headache	Natural labor and 20 min for second stage of labor	LICA	Anticoagulation	Complete recovery
12	^[19]^	41	18	Headache and transient ischemic attack	Not reported	LVA	Not reported	Complete recovery
13		35	5	Headache and cerebral infarction	Not reported	RVA	Not reported	Complete recovery
14		38	8	Headache and Horner syndrome	Not reported	LICA	Not reported	Complete recovery
15		27	11	Headache and neck pain	Not reported	RICA	Not reported	Complete recovery
16		38	7	Headache	Not reported	RVA	Not reported	Complete recovery
17		34	7	Headache and neck pain	Not reported	RVA	Not reported	Complete recovery
18	^[37]^	28	10	Headache and left mild hemiplegia	Prolonged second stage of labor and cesarean section	RICA	Not reported	Not reported
19	^[27]^	35	21	Left hemiplegia	Not reported	BICAs	Not reported	Complete recovery
20	^[23]^	24	14	Headache, right upper limb weakness and facial paralysis	Cesarean section	LICA	Anticoagulation	Complete recovery
21	^[39]^	28	2	Headache	Not reported	LVA	Antiplatelet+anticoagulation	Complete recovery
22	^[17]^	41	6	Headache and subarachnoid hemorrhage	Cesarean section	RICA	Antiplatelet+anticoagulation	Complete recovery
23	^[16]^	43	11	Headache, transient aphasia and mild hemiplegia	Cesarean section	LICA	Antiplatelet	Complete recovery
24		37	15	Headache	Cesarean section	RICA	Antiplatelet	Complete recovery
25	^[36]^	31	5	Neck pain	Cesarean section	BICAs+BVAs	Anticoagulation	Complete recovery
26	^[41]^	32	7	Headache and transient sensory disturbance of left lower limb	Natural labor	RICA	Anticoagulation	Complete recovery
27	^[24]^	37	3	Headache and neck pain	Natural labor	BVAs	Anticoagulation	Complete recovery
28	^[20]^	32	14	Weakness on 1 side, headache, blurred vision	Natural labor and 5 min for the second stage of labor	BICAs	Antiplatelet+anticoagulation	Complete recovery
29	^[63]^	39	10	Headache	Cesarean section	BICAs+BVAs	Anticoagulation	Complete recovery
30	^[29]^	30	5	Headache, neck pain, and blurred vision	Cesarean section	BICAs	Anticoagulation	Complete recovery
31	^[21]^	27	12	Headache	Not reported	LICA	Anticoagulation	Slight inertia
32	^[40]^	31	7	Headache	Natural labor	BICAs+BVAs	Anticoagulation	Not reported
33	^[22]^	35	0	Not reported	Natural labor	BICAs	Anticoagulation	Not reported
34	^[6]^	39	11	Right blepharoptosis, headache, and neck pain	Natural labor and 8 min for the second stage of labor	BICAs+BVAs	Anticoagulation	Complete recovery
35		29	53	Headache, right hemiplegia, loss of sensation and aphasia	Natural labor and 14 min for the second stage of labor	LICA+BVAs	Antiplatelet+anticoagulation+stenting	Complete recovery
36		32	0	Headache and neck pain	Natural labor and 4 h for the second stage of labor	RICA	Antiplatelet +anticoagulation	Left upper limb weakness and facial paralysis
37		39	24	Headache, neck pain, blurred vision, and diplopia	Natural labor and 43 min for the second stage of labor	BVAs	Antiplatelet	Complete recovery
38		28	4	Headache, neck pain, and weakness of both legs	Cesarean section	LICA	Antiplatelet+anticoagualtion+stenting	Complete recovery
39	^[32]^	36	10	Headache	Cesarean section	BVAs	Antiplatelet	Mild right blepharoptosis and facial hypoesthesia
40	^[25]^	35	21	Dizziness	Not reported	RVA	Anticoagulation	Mild right ataxia
41	^[33]^	35	5	Headache	Not reported	RICA	No	Complete recovery
42	^[28]^	29	14	Left hemiplegia	Cesarean section	BICAs	Not reported	Not reported
43	^[38]^	30	6	Headache	Not reported	RVA	Antiplatelet+anticoagulation	Complete recovery
44		30	6	Left neck pain	Not reported	LVA	Antiplatelet	Complete recovery
45	Ours	30	45	Keck pain and left lower limb weakness	Cesarean section	BICAs+BVAs	Antiplatelet	Complete recovery

BICA = bilateral internal carotid artery, BVAs = bilateral vertebral arteries, CN = case number, LICA = left internal carotid artery, LVA = left vertebral artery, RICA = right internal carotid artery, RN = reference number, RVA = right vertebral artery.

All patients underwent imaging examination including head magnetic resonance angiography (MRA), computed tomographic angiography (CTA) and/or digital subtraction angiography (DSA). Among 45 patients, arterial dissections involved unilateral carotid or vertebral artery in 29 cases (64%), bilateral carotid or vertebral arteries in 8 (18%), 3 arteries in 3 (7%), and all bilateral carotid and vertebral arteries in 5 (11%). Fourteen (31%) patients were treated with antiplatelet agents, 27 (60%) with anticoagulation, 7 (16%) with both antiplatelet and anticoagulation medications, and only 2 (4%) with stent angioplasty.

The prognosis was evaluated in 35 patients, including 30 (86%) patients with complete recovery and 5 (14%) with mild focal neurological symptoms which did not affect daily life.

## 4 Case report

**Figure 1:**
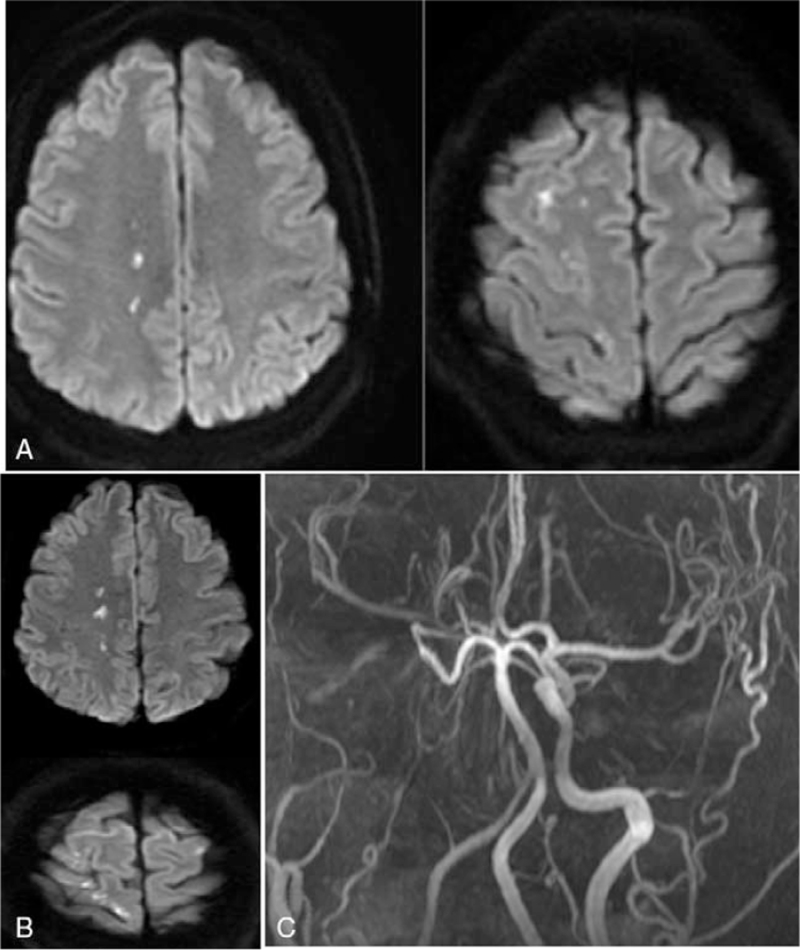
Diffusion weighted imaging (DWI) of the head magnetic resonance imaging (MRI) and angiography (MRA) were performed in the woman before admission. A & B. DWI imaging revealed multiple lesions of acute infarction in the right frontal and parietal lobes. C. MRA showed occlusion of right internal carotid artery.

**Figure 2:**
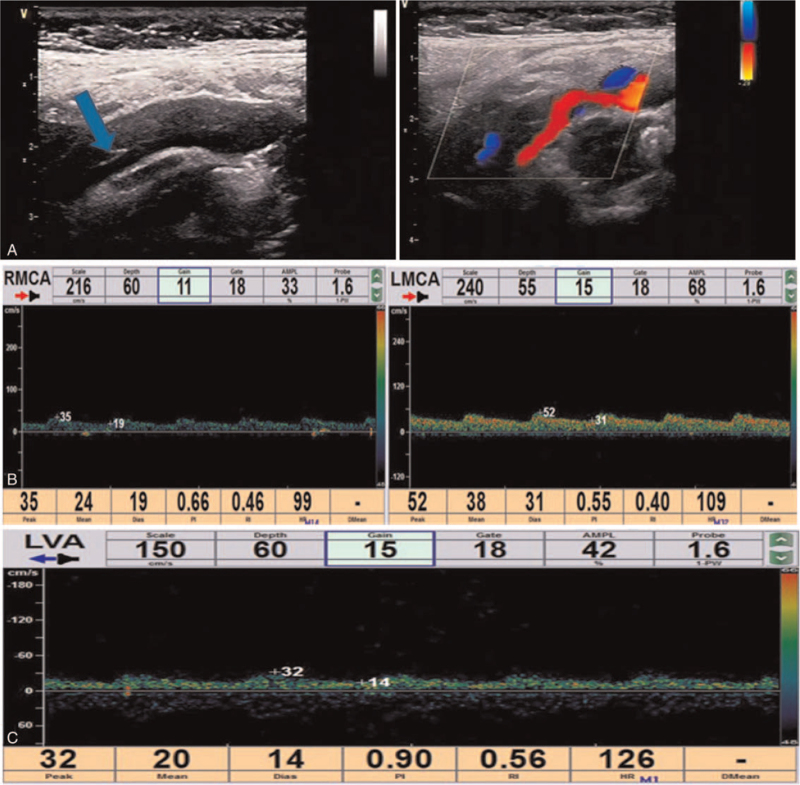
Cervical vascular ultrasound and transcranial Doppler (TCD) examination. A. Cervical vascular ultrasound showed a membrane structure with moderate echo in the lumen of the right common carotid arteries, pulsating with the blood flow. Low echo could be detected in the lumen of the lesion, resulting in local severe stenosis of the common carotid artery and distal occlusion of the internal carotid artery. The initial diagnosis was carotid arterial dissection with intramural hematoma. B & C. The pulsation of bilateral middle cerebral arteries was low, especially on the right side, and the low velocity blood flow signal was detected in the left vertebral artery, with relatively high resistance changes. LVA = left vertebral artery.

**Figure 3:**
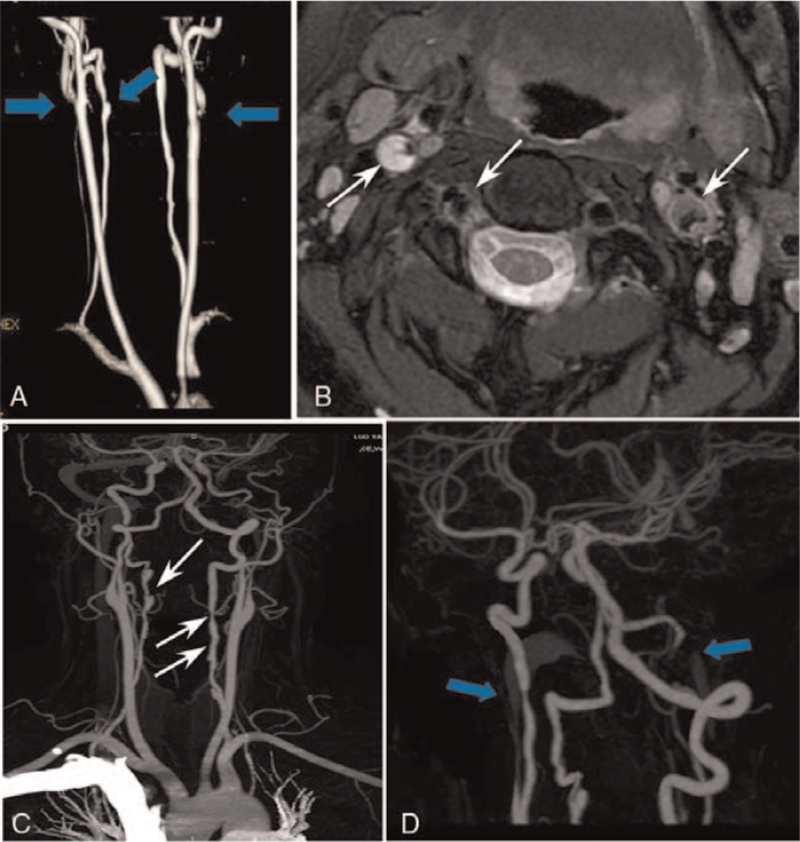
Cervical magnetic resonance angiography (MRA) and head and neck computed tomography angiography (CTA). A & B. Cervical MRA demonstrated stenoses of bilateral carotid and vertebral arteries with crescent signal in the lumen of bilateral carotid arteries and right vertebral artery extracranial segment. C & D. Head and neck CTA displayed diffuse lesions with aneurysm-like changes in bilateral carotid and vertebral arteries.

**Figure 4:**
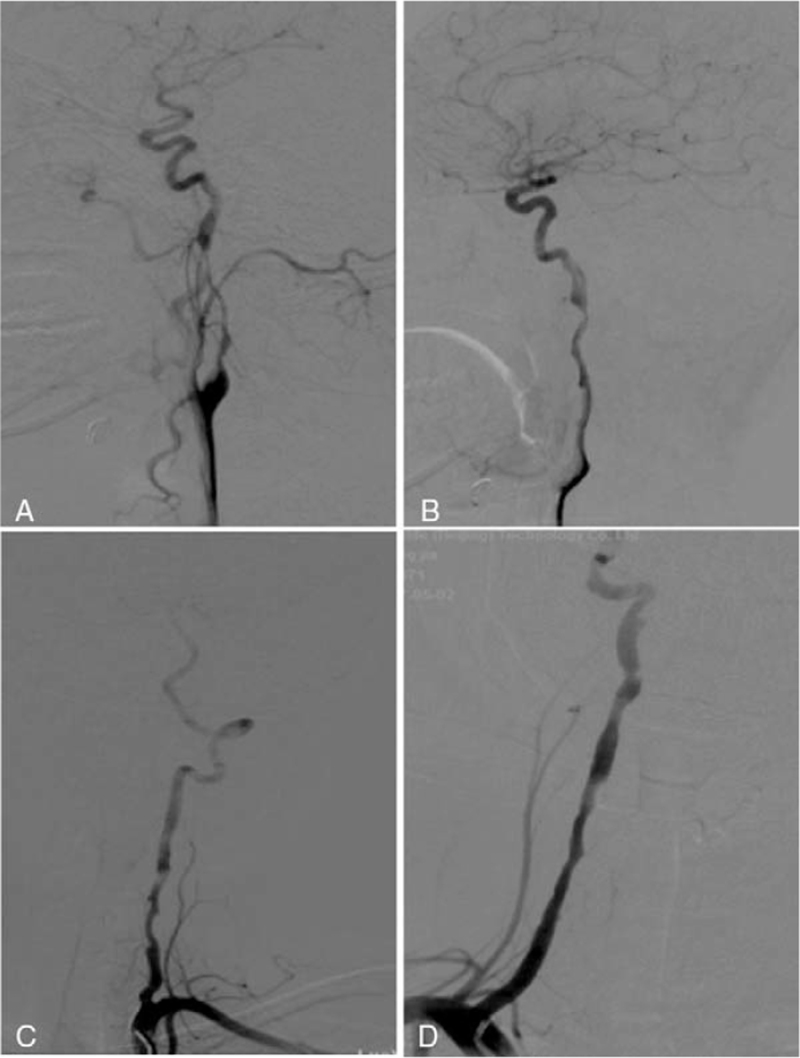
Digital subtraction angiography of the whole brain showed multiple stenoses in right (A) and left (B) internal carotid arteries and right (C) and left (D) vertebral arteries, with poor blood flow to the distal arterial branches.

An otherwise healthy 30-year-old female patient from our hospital was hospitalized because of neck pain and weakness of left leg for 2 days 6 weeks after cesarean delivery of a second baby. Physical examination showed the muscle strength of the left lower limb of grade 4, with the tendon reflex ++. Blood routine, hemagglutination, D-dimer, liver and kidney function test, electrolyte, blood homocysteine, 12 items of immunity, 3 items of vasculitis, thyroid function, glycosylated hemoglobin and C-reactive protein were all normal. The systolic blood pressure was 125 mm Hg, and the diastolic blood pressure was 83 mm Hg. The body mass index (BMI) was normal (21), and no connective tissue disease was present. Magnetic resonance imaging (MRI) revealed multiple acute cerebral infarcts in the right frontal and parietal lobes and right watershed cerebral infarction (Fig. 1) and occlusion of the right internal carotid artery (Fig. 1C). Carotid artery ultrasound (Fig. 2A) revealed a moderate-echo membranous structure pulsating with blood flow in the lumen of the right common carotid artery. Low echo could be detected in the lumen of the lesion, resulting in local severe stenosis of the common carotid artery and distal occlusion of the internal carotid artery. Transcranial Doppler ultrasound (Fig. 2B & C) showed low pulsation of bilateral middle cerebral arteries, especially on the right side, and low velocity blood flow signal was detected in the left vertebral artery, with relatively high resistance changes. Cervical MRA (Fig. 3A & B) demonstrated crescent-shaped signal in the lumen of bilateral internal carotid arteries and right inferior occipital segment of vertebral artery, indicating severe stenosis of bilateral internal carotid arteries. Different signal intensities of the intramural hematomas were shown (Fig. 3B), which indicated that these dissections probably occurred at different time points rather simultaneously, and the only symptomatic dissection was most probably that of the right internal carotid artery. Combined head and neck CTA (Fig. 3C & D) revealed wall thickening of C1 segment of bilateral internal carotid arteries with severe stenosis and multiple aneurysms in the left V1 and V2 segments and right V2 segment of vertebral artery. DSA (Fig. 4) demonstrated a long tubular stenosis with wavy appearance in the right extracranial segment of internal carotid artery (carotid sinus not involved) and a severe stenosis at C1/C2 junction with slow blood flow velocity at the distal end. Multiple stenoses and dissecting aneurysms were also shown in the V2 segment of bilateral vertebral arteries. The initial diagnosis was myofibrous dysplasia because of long stenoses and dissections in bilateral C1 and V2 segments.

**Figure 5:**
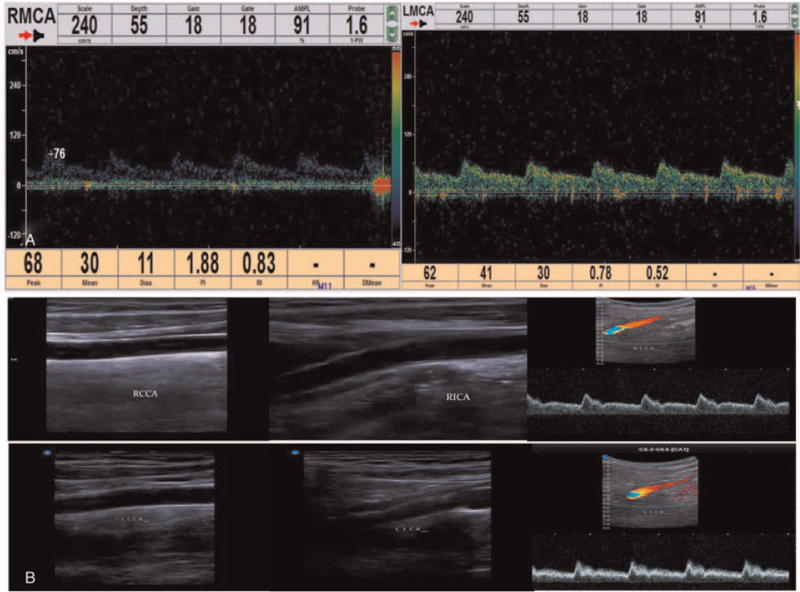
Transcranial Doppler and cervical vascular ultrasound. A. Transcranial Doppler 2 months after discharge demonstrated that the blood flow velocity and pulsatility index in the middle cerebral artery were significantly increased, and the spectrum of intracranial artery was improved. B. Cervical vascular ultrasound 6 months after discharge revealed no vascular abnormality. LMCA = Left middle cerebral artery; RCCA = right common carotid artery; RICA = right internal carotid artery; RMCA = right middle cerebral artery.

**Figure 6:**
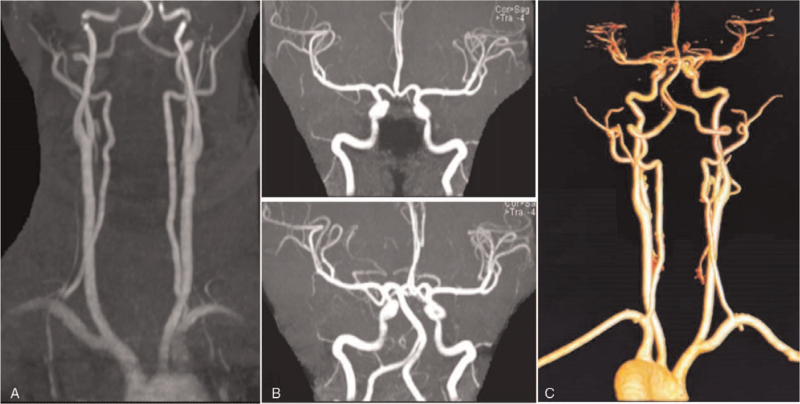
Magnetic resonance angiography (MRA) and computed tomography angiography (CTA). A & B. Head and neck MRA 6 months after discharge revealed nothing abnormal. C. Head and neck CTA 3.5 years after discharge demonstrated nothing abnormal.

After admission, the patient was treated with antiplatelet medications (aspirin enteric coated tablets 0.1 g qd combined with clopidogrel bisulfate tablets 75 mg qd). Neck pain and left lower limb weakness were relieved at discharge 1 week later. Follow-up transcranial Doppler ultrasound 2 months later (Fig. 5A) revealed increases of the middle cerebral artery velocity and pulsatility index and improvement of the spectrum of intracranial artery. Six months later, there was no abnormality in color Doppler ultrasound (Fig. 5B) or MRA of head and neck (Fig. 6A & B). The final diagnosis was postpartum cerebral arterial dissections. Followed up for 3.5 years, the patient was well recovered, with no presence of cerebral arterial dissections on head and neck CTA (Fig. 6C).

## 5 Discussion

In this study, 45 patients with postpartum cerebral arterial dissections were presented including 1 case from our hospital, with the most common symptoms of headache and neck pain, followed by focal neurological symptoms several hours or days later. Most patients had the symptoms within 6 weeks after delivery (mean 11 days). Postpartum cerebral arterial dissections mostly occurred in women with an advanced maternal age (over 30 years), and the most common location involved unilateral carotid or vertebral artery, followed by bilateral carotid or vertebral arteries. The commonest treatment was anticoagulation and antiplatelet medications, and only 2 (4.4%) patients were treated with stent angioplasty. The prognosis was good if managed properly, with complete recovery in 86% and mild focal neurological symptoms in 14%.

Currently, the mechanism of postpartum cerebral arterial dissections is not clear. Although it is generally accepted that connective tissue diseases are closely related to postpartum cerebral arterial dissection, most of these dissections occur in otherwise young healthy women. Tension and Valsalva during delivery may be one of the inducing factors of cerebral artery dissection. However, some women with postpartum cerebral arterial dissections did not have straining or Valsalva action, especially in patients with cesarean delivery.^[26,36]^ Among the 45 patients, 13 cases had only cesarean delivery, and some patients had short second stages of labor. Moreover, the headache and neck pain of postpartum cerebral arterial dissections occurred within 6 weeks (mean 11 days) after delivery, which also indirectly suggests that the tension during delivery and Valsalva movement are not entirely the cause of cerebral arterial dissection. In our study, the case was initially diagnosed with fibromuscular dysplasia because of long stenosis and dissections in bilateral carotid and vertebral arteries, however, follow-up imaging 6 months after discharge cleared the mis-diagnosis because all the vascular lesions had been completely resolved. A final diagnosis of postpartum cerebral arterial dissection was drawn. Research of the literature revealed that fibromuscular dysplasia had rarely associated with postpartum cerebral arterial dissection, and the most definitive association between fibromuscular dysplasia and pregnancy is spontaneous coronary arterial dissection, which may cause acute coronary syndromes, cardiac arrest, and sudden death of women with fibromuscular dysplasia in the postpartum period.^[44–46]^ Fibromuscular dysplsia is a noninflammatory, nonatheroslerotic vascular disorder affecting primarily the renal and extracranial carotid and vertebral arteries.^[47]^ Fibromuscular dysplasia could be found in approximately 15% of patients with a spontaneous dissection of the carotid or vertebral arteries, and may cause spontaneous arterial dissections, intracranial aneurysms, arterial redundancies, widened aortic root, and increased arterial distensibility.^[48]^ Hyperhomocysteinemia was not found to associate with postpartum cervical arterial dissections, however, hyperhomocysteinemia may be a risk factor for cervical arterial dissections.^[49–51]^

In as early as 1983, Wiebers^[52]^ had pointed out that pregnancy (including prenatal and postpartum periods) increased the incidence of cerebral infarction up to 13 times compared with patients without pregnancy. In the study of association between pregnancy and cervical artery dissection by Omran et al,^[53]^ it was found that pregnancy was associated with a higher risk of cervical artery dissection in 826 women with pregnancy in comparison with 826 matched control women with renal colic and that arterial dissection might lead to stroke in women with pregnancy, with the increased risk limited to the postpartum period. Kelly et al^[6]^ believed that transient abnormality of vascular walls caused by postpartum hormone changes may be the inducing factor of cerebral arterial dissection. Sudden decreases in postpartum estradiol and progesterone can directly affect the integrity of vascular wall.^[54]^ Hormone-mediated changes of vasoactive substances and degradation of collagen may increase the susceptibility of arterial injury, whereas hypercoagulable state after pregnancy also promotes development of thrombosis at the dissection site. Physiological changes caused by pregnancy, including elevation in blood volume and cardiac output, may promote the pressure of vascular wall, and the high blood pressure on vascular wall increased in preeclampsia, in particular, is considered to be related to arterial dissection because they can increase the stress on intimal linings in high-flow arteries.^[16,19,20,41]^ Actually, postpartum cerebral arterial dissections occur most frequently in normotensive patients, and among 27 patients with these dissections reported in the study by Kelly et al,^[6]^ only 8 (30%) were involved with increased blood pressure at the time of presentations. Furthermore, postpartum cerebral arterial dissections were mostly presented in women of advanced maternal age, with 78% (n = 35) of patients over the age of 30 years (mean 35), which is related to an increased risk of atherosclerosis and arterial stiffness. Currently, it is impossible to identify patients at early risks of postpartum cerebral arterial dissections by recognizing the risk factors because most patients with these risk factors do not have these dissections.

Once there are severe postpartum headache and neck pain, it is necessary to identify postpartum cerebral arterial dissection as soon as possible, and head and neck imaging examination is generally sufficient for a clear diagnosis, with the imaging presentation similar to that of other cerebral arterial dissection. At present, there are no unified imaging diagnosis standards, and it is recommended to use a variety of imaging methods to evaluate the signs of arterial wall and lumen to make clear the diagnosis of cerebral arterial dissection. The diagnosis was made if any one of the following signs was present^[55–57]^: double lumen sign, intimal flap sign, and intramural hematoma on CTA and/or MRA, or reverse flow signal, double lumen change and/or floating intima on ultrasound. The high resolution MRI tube wall imaging technology has been reported to have a high diagnostic efficiency for cerebral arterial dissections and can be used directly to display the characteristics of vascular wall while being non-irradiating and noninvasive.^[58]^ Cerebral MRI with axial T1 Fat-Sat and T2 gradient echo (T2∗) which is highly sensitive to clot and blood should be performed in patients with suspected cerebral venous thrombosis and cervical arterial dissection, both of which can cause postpartum headache complicated by seizures and stroke. Use of this technique^[41,59]^ has resulted in successful detection of difficult cases with cervical carotid arterial dissection.

There were significant differences between the predilection sites of cerebral artery dissection and atherosclerosis. Carotid arterial dissection often occurs in C1 segment (carotid segment), with the distal part of internal carotid artery system being often involved, whereas atherosclerosis is easy to involve the beginning of carotid artery.^[60]^ For the posterior circulation system, vertebral arterial V1 and V3 segments (57%) were the most common sites of dissection, and multi-segment dissection accounted for 16%,^[61]^ which may be related to the lack of bone structure support and high activity around the above sites. The prone sites of atherosclerosis often involve the origin of vertebral artery and the junction of vertebral basilar artery.^[62]^

Unilateral lesions were more common in patients with cerebral arterial dissection, whereas bilateral lesions were rare, especially in bilateral carotid and vertebral arteries. The patient from our hospital was the fifth to involve bilateral carotid and bilateral vertebral arteries,^[34,36,40,63]^ which was confirmed by MR angiography, CTA, and DSA. The first case of postpartum cerebral arterial dissections involving bilateral carotid and bilateral vertebral arteries was reported in 2003 by Oehler et al^[34]^ Although postpartum cerebral arterial dissection is rare, delayed diagnosis and treatment may lead to severe and irreversible neurological deficits.

Postpartum cerebral arterial dissection rarely relapses and can take place in the first 2 months after onset, which indirectly supports the cause of transient vascular disease.^[54]^ Recurrence can occur if early treatment is not timely. Poor prognosis is mainly related to missed diagnosis and delayed treatment.

At present, there is no unified standard for the treatment of postpartum cerebral artery dissection. The drugs used in the literature include antiplatelet aggregation, anticoagulant therapy, and endovascular stent implantation in only a few cases. The prognosis of the disease is relatively good, and only 5 cases (14%) have mild symptoms. In our study enrolling patients with cerebral or intracranial arterial dissections in the postpartum period, the patient was treated with antiplatelet aggregation therapy after admission, with good prognosis and no sequelae. In the CADISS randomized clinical trial investigating antiplatelet vs anticoagulation therapy in 255 patients with extracranial cervical carotid and vertebral arterial dissections,^[64]^ the recurrence at 1 year follow-up was low (2.4% or 2.5%), and no significant (*P* > .05) difference was found between treatment groups in outcome events or the rate of recanalization. In the TREAT-CAD randomized and non-inferiority trial investigating the effect of aspirin vs anticoagulation on cervical arterial dissection in 194 patients,^[65]^ it was found that aspirin was non-inferior to vitamin K antagonists in the treatment of cervical arterial dissection. This study had some limitations, including the retrospective and single-center design, presentation with few cases, and a greater heterogeneity caused by cases from different races and areas, which may all affect the outcomes. Moreover, since carotid arterial dissection associated with childbirth is very rare, it is probable that not many patients can be enrolled in 1 single center. Detailed case description and reports should be presented in open-published sources for reference.

In summary, postpartum cerebral arterial dissections mainly present within 6 weeks after delivery in women of an advanced maternal age, and the clinical symptoms are not typical with early presentation of headache which can easily be misdiagnosed. Differentiation diagnosis should be made from venous sinus thrombosis, myofibrous dysplasia, and reversible cerebral vasoconstriction syndrome. Prognosis is usually favorable in patients with early diagnosis and prompt treatment. Clinicians should improve their understanding of headache in patients with unclear etiology. In addition to routine MRI and MRA, neck vascular color Doppler ultrasound and neck MRA should also be performed in order to exclude postpartum cerebral artery dissection.

## Author contributions

**Data analysis:** Chun-Yun Ruan, Bu-Lang Gao.

**Data collection:** Chun-Yun Ruan, Hong-Li Pang, Kun Zhang, Yao-Hui Zhang, Li-Ping Wei.

**Literature research:** Chun-Yun Ruan, Hong-Li Pang, Yao-Hui Zhang.

**Revision of the original version:** Bu-Lang Gao.

**Study design:** Bu-Lang Gao, Zi-Liang Wang.

**Supervision:** Li-Ping Wei.

**Writing of the original version:** Chun-Yun Ruan.
